# Hyperbaric Oxygen Therapy for Branch Retinal Artery Occlusion: A Case Series

**DOI:** 10.7759/cureus.96804

**Published:** 2025-11-13

**Authors:** Mateusz Zarzecki, Izabela Zawadzka, Blanka Mitera, Marzena Wojewódzka-Żelezniakowicz, Joanna Konopińska

**Affiliations:** 1 Department of Ophthalmology, Medical University of Bialystok, Bialystok, POL; 2 Department of Emergency Medicine, Medical University of Bialystok, Bialystok, POL

**Keywords:** branch retinal artery occlusion, central retinal artery occlusion, hemiretinal artery occlusion, hyperbaric oxygen therapy, ophthalmic artery, retinal artery occlusion, visual acuity

## Abstract

Retinal artery occlusion (RAO) is an ophthalmic emergency primarily caused by systemic cardiovascular diseases. Central RAO (CRAO) may lead to severe visual loss, whereas branch retinal artery occlusion (BRAO) is associated with more benign symptoms and often resolves with less severe visual dysfunction. The etiopathogenesis of RAO is similar to that of ischemic stroke. It involves sudden occlusion of the retinal artery, leading to ischemia of the inner layers of the retina, retinal infarction, permanent damage to sensory cells, and resultant irreversible loss of vision. Although small-sample studies on the treatment of CRAO are available, only a few case series and reports on the efficacy of hyperbaric oxygen therapy (HBOT) in BRAO have been published. In this case series, we aimed to evaluate the effectiveness of HBOT in improving visual acuity in patients with BRAO based on an analysis of selected case reports. Patients underwent HBOT at 5, 36, and 48 hours after the onset of ocular symptoms, and visual acuity improved both quantitatively and qualitatively. HBOT has beneficial effects on the final visual acuity of patients following an episode of BRAO and may be a rescue treatment for early-stage BRAO, particularly when reperfusion is still feasible.

## Introduction

Retinal artery occlusion (RAO) is the complete or partial obstruction of the ophthalmic artery that can induce severe ischemia of the affected eye and associated ocular tissues [1]. Based on its location, RAO can be classified into central retinal artery (CRA) occlusion (CRAO), branch retinal artery occlusion (BRAO), temporal and nasal branches of the superior CRA hemiretinal artery occlusion (HRAO), and cilioretinal artery occlusion (CLRAO) [2]. CRAO and BRAO are the most common forms of RAO. However, BRAO is more prevalent than CRAO, with incidence rates of approximately 5 and 1-2 cases per 100,000 people per year, respectively [3]. The incidence of RAO differs among various populations [4]. The etiopathogenesis resembles that of ischemic stroke and involves the sudden occlusion of the retinal artery by cholesterol or platelet-fibrin emboli secondary to carotid or aortic atherosclerosis. The resulting ischemia of the inner retinal layers, along with retinal infarction and permanent damage to sensory cells, causes irreversible vision loss [3]. Additionally, inflammatory and autoimmune factors have been implicated in the etiology of RAO [3,5]. Visual outcomes after CRAO range from light perception to hand movement and may differ based on the presence of a cilioretinal artery (present in 15-30% of the population), which may facilitate better post-CRAO visual acuity. Following symptom onset, most patients with BRAO have a best-corrected visual acuity (BCVA) of 0.1. In 60-89% of cases, the final BCVA improved to 0.5 in one study [4]. BRAO-associated reduction in BCVA is painless and can result in sectoral visual field defects with a commensurate BCVA loss depending on the vessel involved [6]. The natural course of BRAO is characterized by variable outcomes: it may resolve spontaneously [7] or lead to complications such as neovascularization of the iris, retina, and optic disc, which occurs in up to 15% of cases [8,9]. The effectiveness of standard conservative treatments such as eye massage, sublingual isosorbide diazotate administration, anterior chamber paracentesis, systemic pentoxifylline administration, and intravenous administration of acetazolamide and mannitol has not been confirmed [10]. The search for new therapies for this disease is currently ongoing due to the lack of a clear therapeutic protocol for its management and effective treatment [11], and newer approaches, such as laser or surgical management, have been proposed. Recently, novel perspectives have indicated a potential adjunctive therapy involving hyperbaric oxygen therapy (HBOT) [1].

HBOT involves inhaling almost pure oxygen in a hyperbaric chamber under a pressure of at least one atmosphere absolute (ATA). This treatment replaces nitrogen in the vitreous body with oxygen, creating an oxygen reservoir lasting for up to 48 hours post-therapy [12]. Experimental studies using animal models have shown that HBOT can potentially protect ischemic retinal neurons from apoptosis. Positive effects of HBOT in the treatment of retinal ischemia have also been observed in human patients. The therapy enhances the diffusion gradient from the choroidal circulation to the outer retinal layers and facilitates the adequate transfer of supplemental oxygen from the outer to the inner retinal layers [1,2]. This effect protects the injured retinal neuronal cells from apoptosis and maintains retinal viability during the reperfusion period in patients with RAO.

Small-sample studies on the treatment of CRAO are available [13-16], but only a few case series and reports on the efficacy of HBOT in BRAO have been published. Schmidt et al. [17] compared the clinical outcomes of 17 patients with non-arteritic BRAO who underwent HBOT to those of 160 patients who received conservative treatment. The final BCVAs of the HBOT and control groups were 0.69 ± 0.29 and 0.32 ± 0.23, respectively (p < 0.05). No adverse events occurred in any of the patients who underwent HBOT. Demir et al. [18] described the case of a healthy 20-year-old woman who experienced a sudden decrease in BCVA due to a BRAO-associated visual field defect in the right eye. After 10 HBOT sessions, the visual acuity was restored to 20/25. A 45-year-old man with concurrent BRAO and branch retinal vein occlusion (BRVO) was successfully treated using 20 HBOT sessions [19], which improved the initial BCVA of 20/200 to 25/25 after five months of follow-up. BRAO may not be vision-threatening, but it can substantially worsen the quality of vision due to visual field defects. In cases of BRAO in monocular patients, achieving the highest possible BCVA is crucial. Additionally, young patients may benefit from adjunctive HBOT.

In this case series, we aimed to highlight the potential benefits of HBOT for patients with BRAO. The insights from the cases will contribute to improving BRAO management, influence healthcare policy, and guide future research to underscore the significance of HBOT.

## Case presentation

Case 1

**Figure 1:**
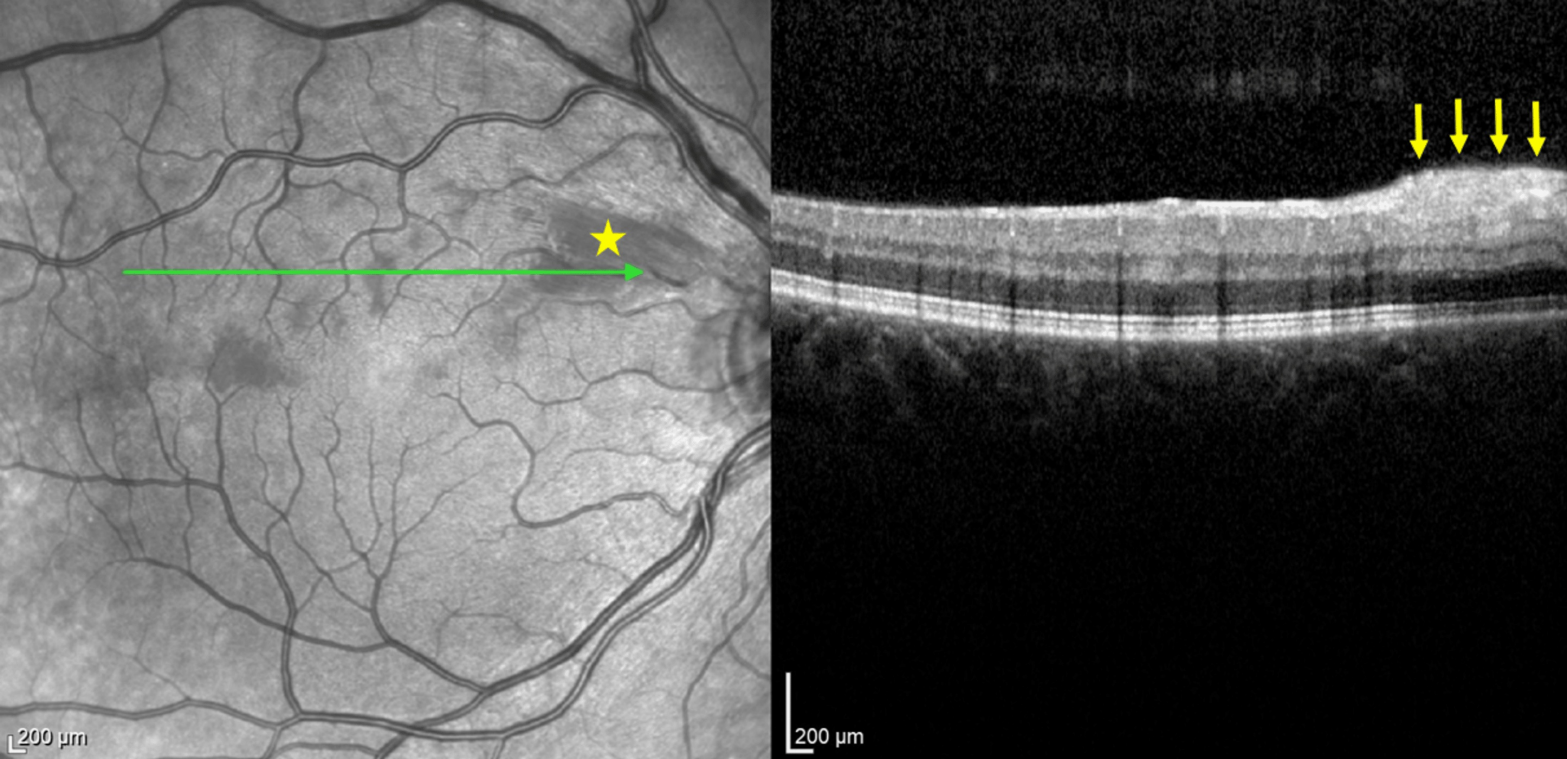
OCT image of the retinal layers of the papillomacular bundle at the initial visit. The yellow star indicates the area of swelling of the RNFL. Yellow arrows indicate edema of the inner and middle retinal layers. OCT, optical coherence tomography; RNFL, retinal nerve fiber layer.

**Figure 2:**
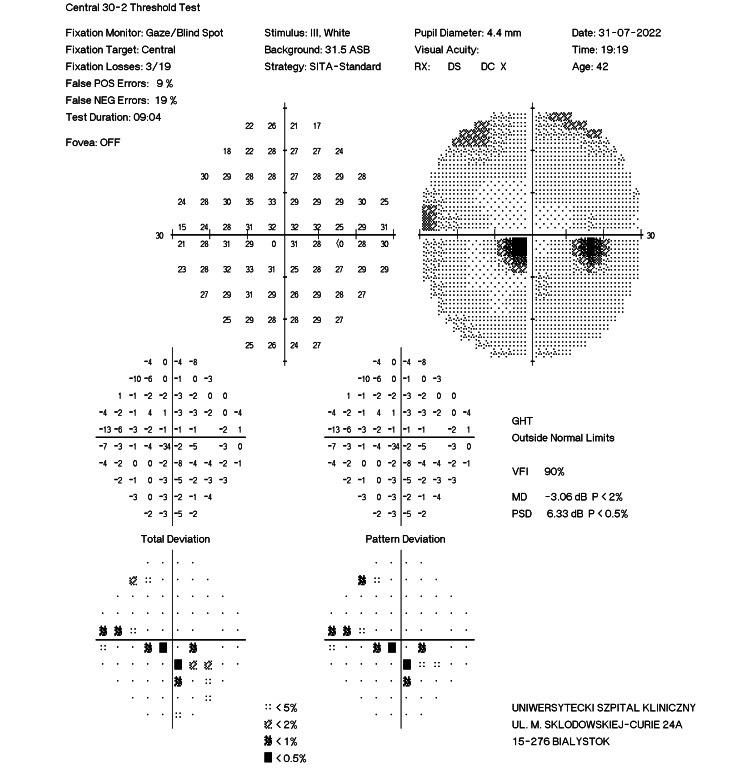
Perimetry of the right eye of case 1 (before treatment).

A 42-year-old woman presented to the Department of Ophthalmology with visual field loss below the visual axis of the right eye, which she first noticed upon waking. She felt no pain and had no redness or other ophthalmic symptoms. There was no history of systemic disease or chronic drug treatment. On examination, the BCVA of the right and left eyes was 0.8 and 1.0, respectively. The bilateral intraocular pressure (IOP) was within the normal limit (18.0 mmHg) when tested using the Goldmann applanation tonometer (GAT). No impairment in color discrimination was detected with the Ishihara plates test. A bilateral anterior segment ophthalmic examination revealed no abnormality. Examination of the right fundus revealed a normal optic nerve disc. However, peripapillary swelling of the nerve fibers in the superior temporal quadrant that extended along the superior choroidal arcade toward the fovea was observed, along with a single intraretinal hemorrhage on the temporal side. Optical coherence tomography (OCT; Heidelberg Engineering OCT Spectralis®, Heidelberg, Germany) of the optic disc and macula revealed swelling of the retinal nerve fiber layer (RNFL) in the temporal and superior temporal quadrants, with corresponding edema of the inner and middle retinal layers involving the papillomacular bundle (PMB) (Figure 1). Perimetry (30-2 SITA Standard visual field test) of the right eye revealed a paracentral scotoma predominantly in the superior hemisphere (mean deviation (MD), −3.06 dB) (Figure 2). No abnormality was detected by laboratory tests or computed tomography (CT) of the brain or craniofacial regions. Inflammatory processes were ruled out based on negative C-reactive protein (CRP) and erythrocyte sedimentation rate (ESR) results (Table 1).

**Table 1:** Laboratory tests results (case 1). CRP, C-reactive protein; ESR, erythrocyte sedimentation rate.

Laboratory test	Test results	Reference range
CRP	<1.0 mg/L	0.0-10.0 mg/L
ESR	22 mm/2 h	<24 mm/2 h

**Figure 3:**
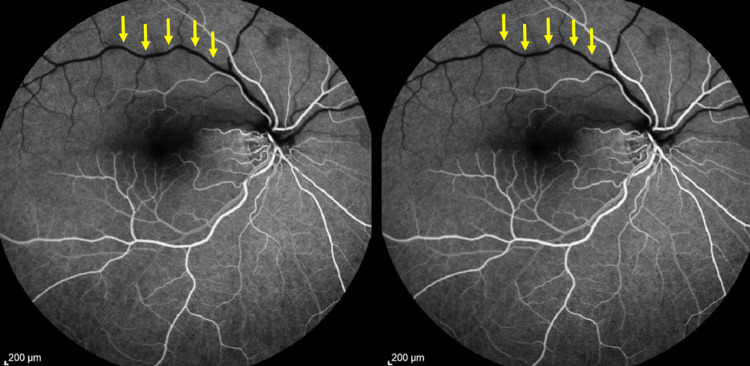
FA images before treatment. Yellow arrows indicate impaired flow in the upper arcade with delayed venous filling in the initial phases of the study. FA, fluorescein angiography.

We expanded the diagnostics with fluorescein angiography (FA), which revealed markedly impaired flow in the upper arcade with delayed venous filling in the initial phases of the study (Figure 3). There was also a particular impairment in the perfusion of the small parafoveal branch and a significant delay in the filling of the unilinear venous branch.

**Figure 4:**
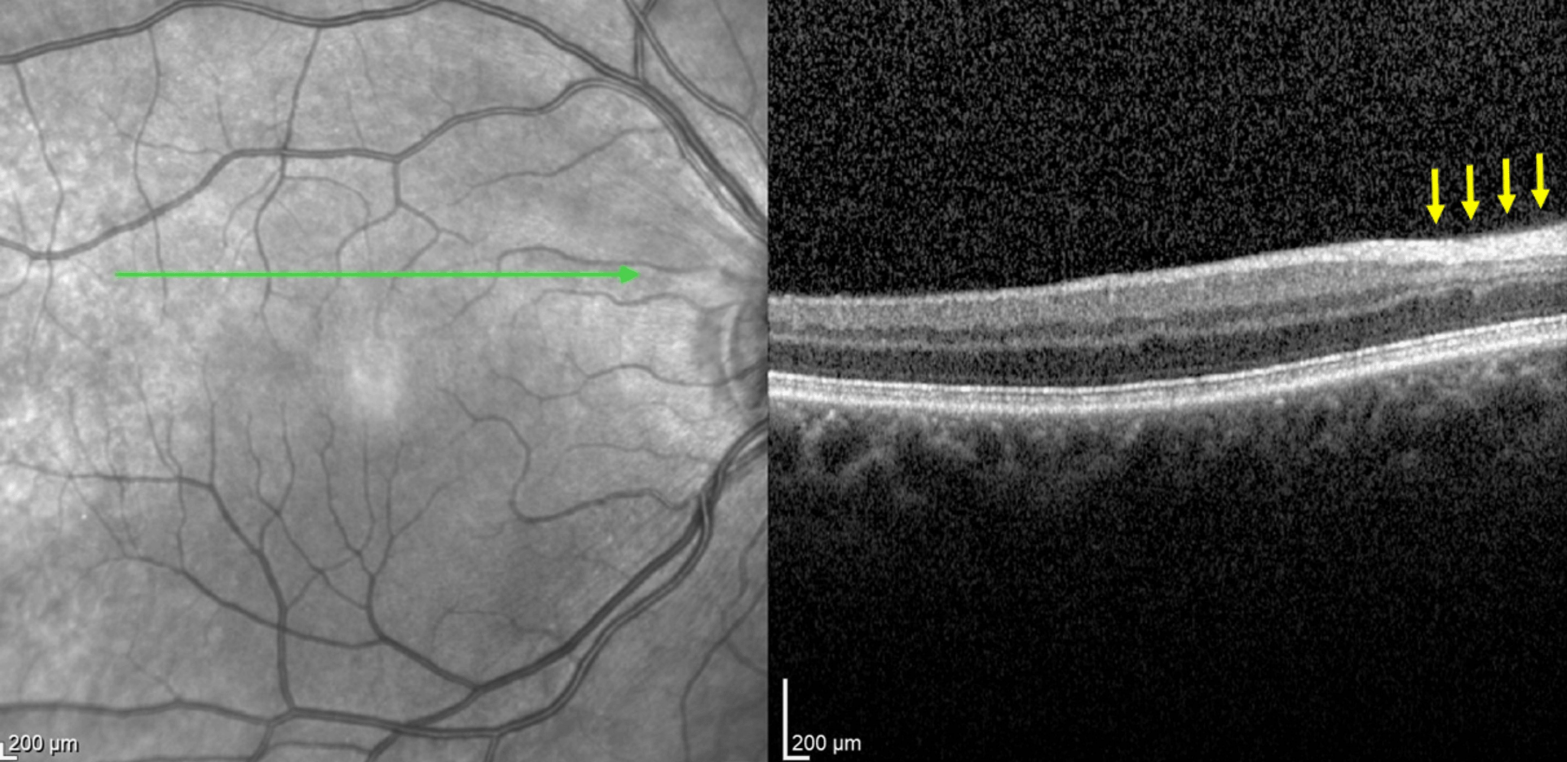
OCT image of the retinal layers of the papillomacular bundle after 10 HBOT sessions. Yellow arrows indicate the post-treatment area of the previous inner and middle retinal layers swelling corresponding to Figure 1. HBOT, hyperbaric oxygen therapy; OCT, optical coherence tomography.

**Figure 5:**
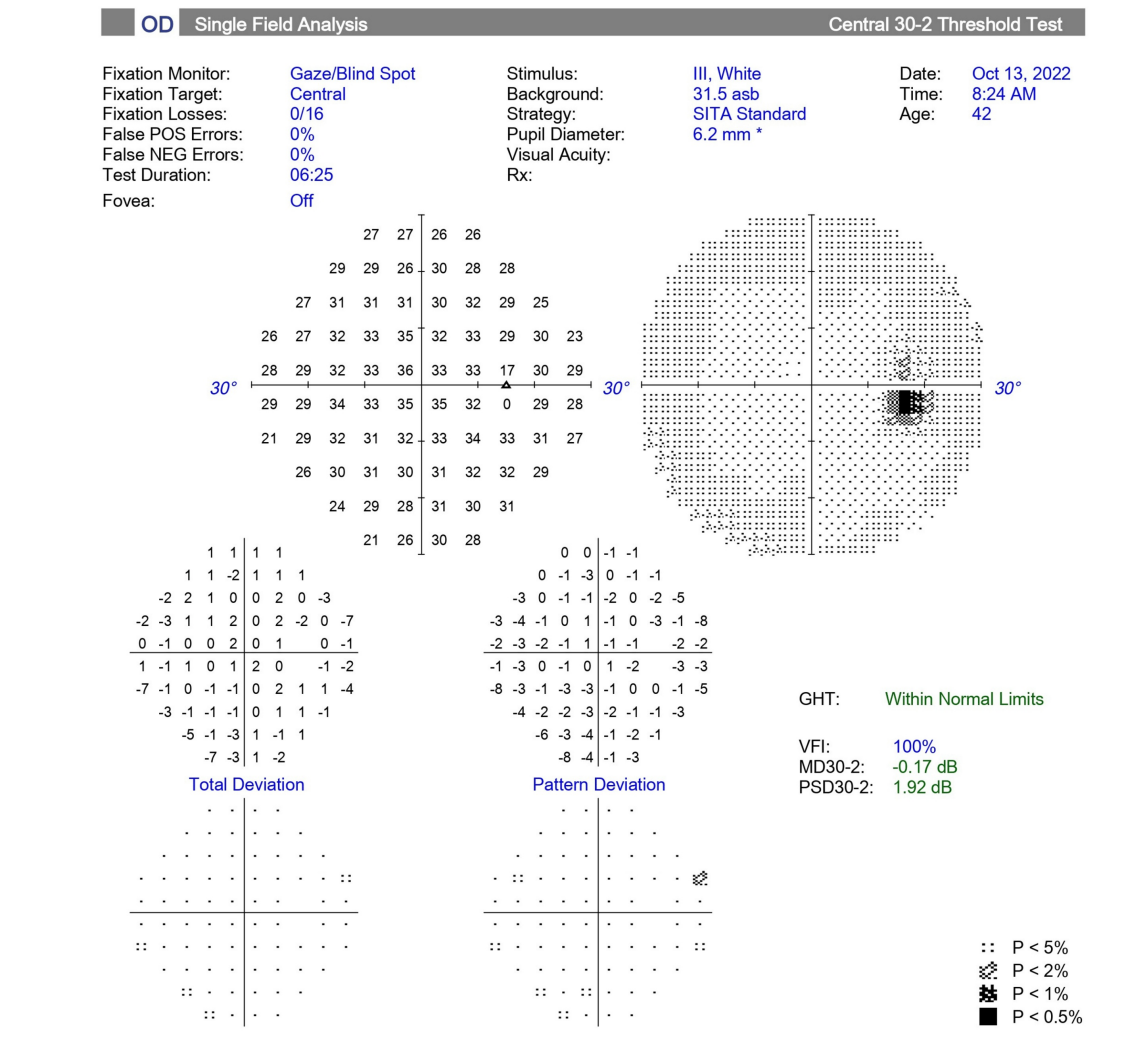
Post-treatment perimetry of the right eye of case 1 (after treatment).

Based on the examinations, we diagnosed paracentral acute middle maculopathy (PAMM) secondary to HRAO of the temporal and nasal superior branches, with significantly impaired perfusion of the small parafoveal branch from the superior temporal artery. The patient was immediately referred for HBOT, and pentoxifylline 400 mg p.o. was prescribed twice daily. The first session of a daily HBOT regimen (1.5-hour HBOT at a compression of 2.5 ATA) in the hyperbaric chamber commenced approximately 36 hours after symptom onset, and the patient completed a total of 13 sessions. The patient reported the disappearance of the scotoma and return of normal vision in the right eye (BCVA 1.0) during follow-up visits on days 7 and 14 after HBOT initiation. Fundus examination and OCT revealed a gradual reduction of swelling in the inner and middle retinal layers (Figures 4, 5), and this trend continued at the one- and three-month follow-up visits after HBOT, when the normal morphological and functional status of the right retina was finally restored. The patient reported no adverse effects or complications, including those related to HBOT or pentoxifylline administration, during or after the treatment period.

Case 2

A 22-year-old woman presented to the hospital outpatient clinic with complaints of glare in the central visual field of the left eye, which had been present for 24 hours. She had previously experienced severe tension-type occipital pain that was refractory to analgesic treatment for three days, as well as periodic, transient scotomas in the visual field of the left eye during the previous year. Her medical history included bipolar affective disorder and gastroesophageal reflux disease, but no family history of chronic diseases. The patient denied regular use of general medications.

**Figure 6:**
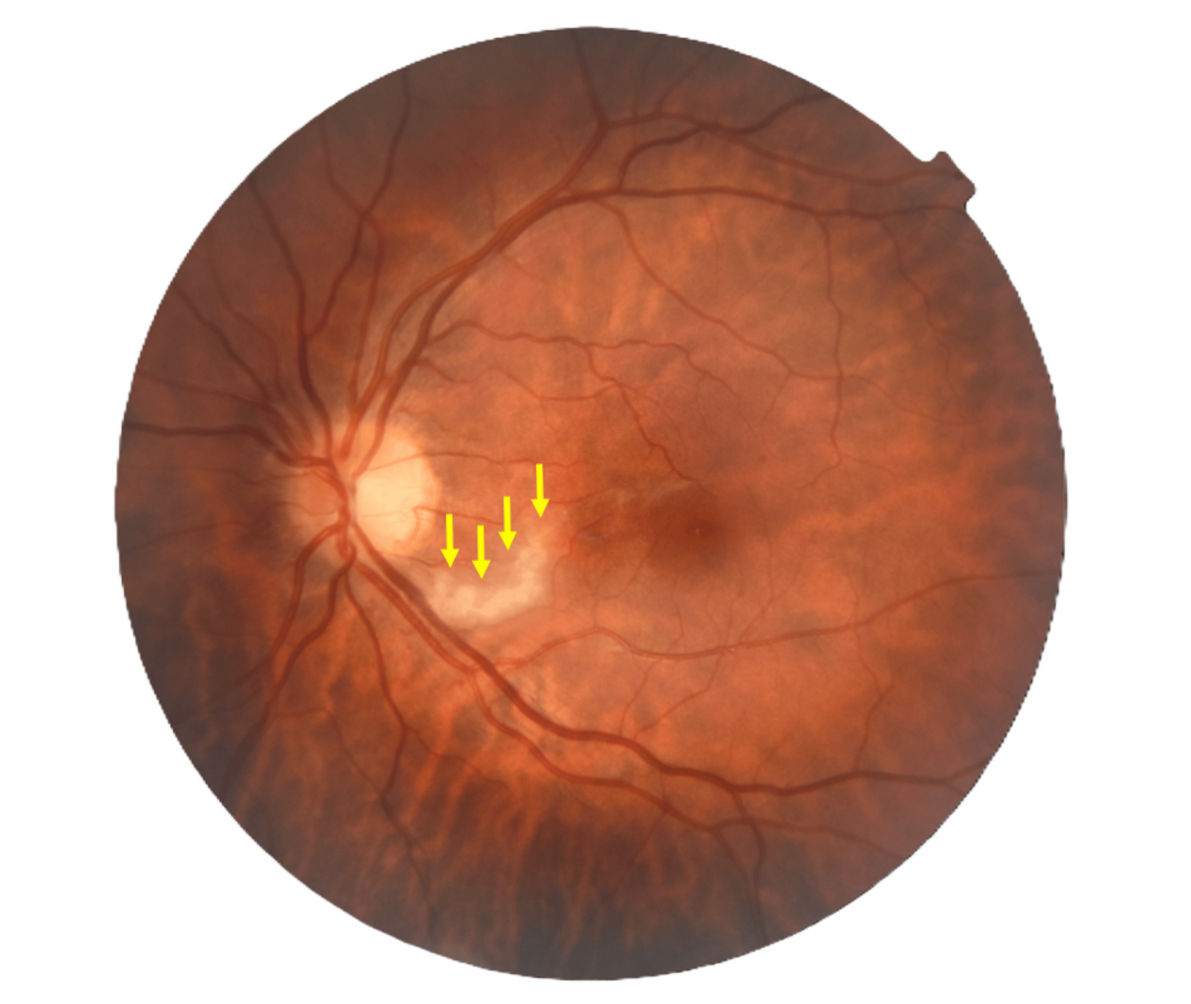
Pretreatment fundus image of the left eye of case 2 at the initial visit (before treatment). Yellow arrows indicate an arched, vaguely circumscribed region of retinal translucency.

The patient underwent both basic and detailed ophthalmological examinations. The BCVA of both eyes was 1.0. The GAT IOPs were 13.5 mmHg and 15.5 mmHg for the right and left eyes, respectively, with normal color vision. Bilateral anterior segment examination revealed no pathological changes. Slit-lamp examination of the left fundus showed a flat, pink optic disc with a slightly blurred nasal border and peripapillary chorioretinal atrophy extending toward the temporal side. This area also contained an arched, vaguely circumscribed region of retinal translucency (approximately 0.5 disc diameter (DD)) at the orifice of the inferior arcade in the PMB (Figure 6). We also observed a dark red, flat choroidal hemangioma-like lesion (approximately 2 DD) on the intermediate retinal periphery in the superior nasal quadrant, with no other pathological changes or abnormalities in the right fundus.

**Figure 7:**
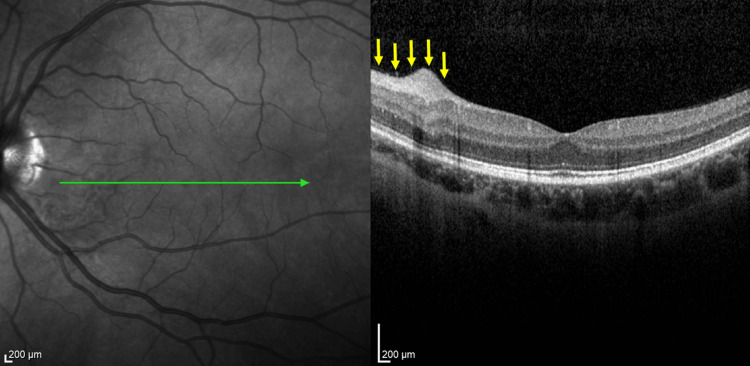
OCT image of the foveal profile with swelling of the inner retinal layers. Yellow arrows indicate the area of swelling of the inner retinal layers. OCT, optical coherence tomography.

**Figure 8:**
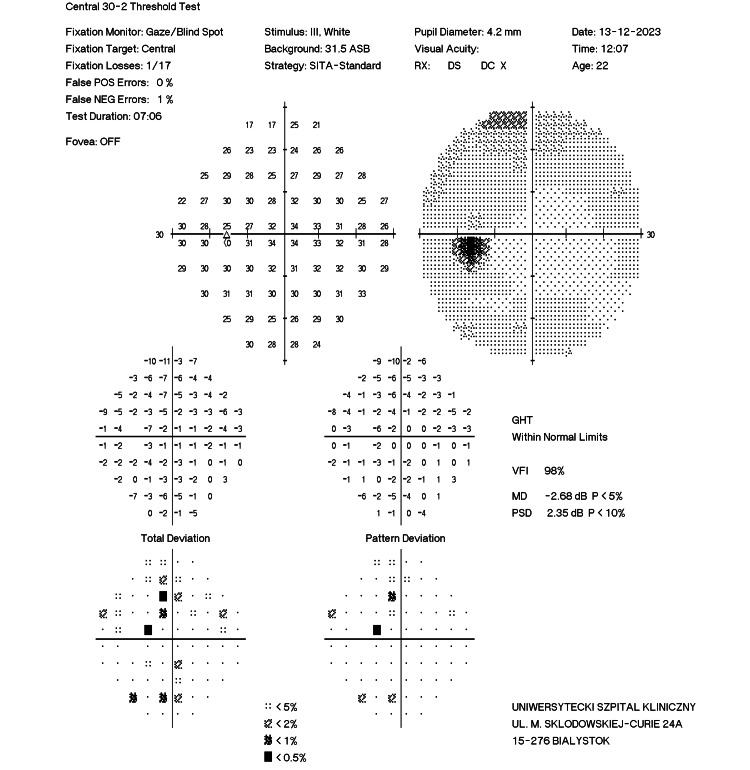
Pretreatment perimetry of the left eye of case 2 (before treatment).

Additional diagnostic studies included an OCT examination (Heidelberg Engineering OCT Spectralis®) of the left macula. The foveal profile was preserved, and there was swelling of the inner retinal layers located temporally and inferiorly from the optic disc (Figure 7). At the intermediate retinal periphery, choroidal vasodilation was observed in the superior temporal quadrant without other abnormalities. Visual field testing (30-2 SITA Standard visual field test) of the left eye revealed characteristic diffuse scotomas predominantly in the upper hemisphere (MD, −2.68 dB) (Figure 8). FA of the left eye showed normal fluorescence temporally from the optic disc, as well as a small arched hypoperfused area at the orifice of the inferior arcade that was not enhanced during the examination. The macular and retinal peripheries showed no pathological leakage of fluorescein dye. The results of all examinations were normal for the right eye. Non-contrast-enhanced CT of the head and craniofacial regions revealed no pathological changes. A wide panel of blood tests for rheumatoid, autoimmune, and infectious diseases was performed. Inflammatory etiology was excluded based on negative CRP and ESR results (Table 2).

**Table 2:** CRP and ESR tests results (case 2). CRP, C-reactive protein; ESR, erythrocyte sedimentation rate.

Laboratory test	Test results	Reference range
CRP	<1.0 mg/L	0.0-10.0 mg/L
ESR	21 mm/2 h	< 24 mm/2 h

Based on these findings, BRAO in the left eye was diagnosed. We immediately referred the patient for HBOT and prescribed pentoxifylline 400 mg orally twice daily. The patient was also referred to a vascular outpatient clinic, and a cardiac echocardiogram was performed. The first session of a daily HBOT regimen (1.5 hours daily at a compression of 2.5 ATA) commenced less than 48 hours after symptom onset. At a follow-up visit three days after HBOT initiation, the patient reported reduced glare intensity, and the BCVA and IOP of the left eye were 1.0 and 9.5 mmHg, respectively. During the fundus examination, we observed fading and sharpening of the borders of the retinal edema zone.

In a transesophageal cardiac echocardiogram performed in the outpatient setting, individual contrast bubbles were observed passing into the left atrium, likely through anastomoses, during the late phase of the Valsalva test. Laboratory tests revealed elevated concentrations of total cholesterol and N-terminal pro-B-type natriuretic peptide. Detailed laboratory tests for autoimmune diseases confirmed positivity for anti-Jo1, anti-Sm, and anti-SSA/Ro antibodies, along with clinically relevant titers of anti-CENP-B, anti-SSB/La, and anti-histone antibodies (Table 3). Based on our previous observations, we prescribed 1,000 mg of citicoline (oral solution) daily.

**Table 3:** Laboratory tests results (case 2). NT-proBNP, N-terminal pro-B-type natriuretic peptide; anti-Jo1, anti-nuclear antibody; anti-Sm, anti-Smith antibody; anti-SSA/Ro, anti–Sjögren's-syndrome antigen A antibody; anti-CENP-B, anti-Centromere Protein B antibody; anti-SSB/La, anti–Sjögren's-syndrome antigen B antibody.

Laboratory test	Test results	Reference range
Total cholesterol	212 mg/dL	<190 mg/dL
NT-proBNP	126.1 pg/mL	0.0-125.0 pg/mL
Anti-Jo1	0.32 kU/L	< 0.3 kU/L
Anti-Sm	0.74 kU/L	< 0.3 kU/L
Anti-SSA/Ro	0.33 kU/L	< 0.3 kU/L
Anti-CENP-B	1.40 kU/L	< 0.3 kU/L
Anti-SSB/La	1.40 kU/L	< 0.3 kU/L
Anti-histone	0.92 kU/L	< 0.3 kU/L

**Figure 9:**
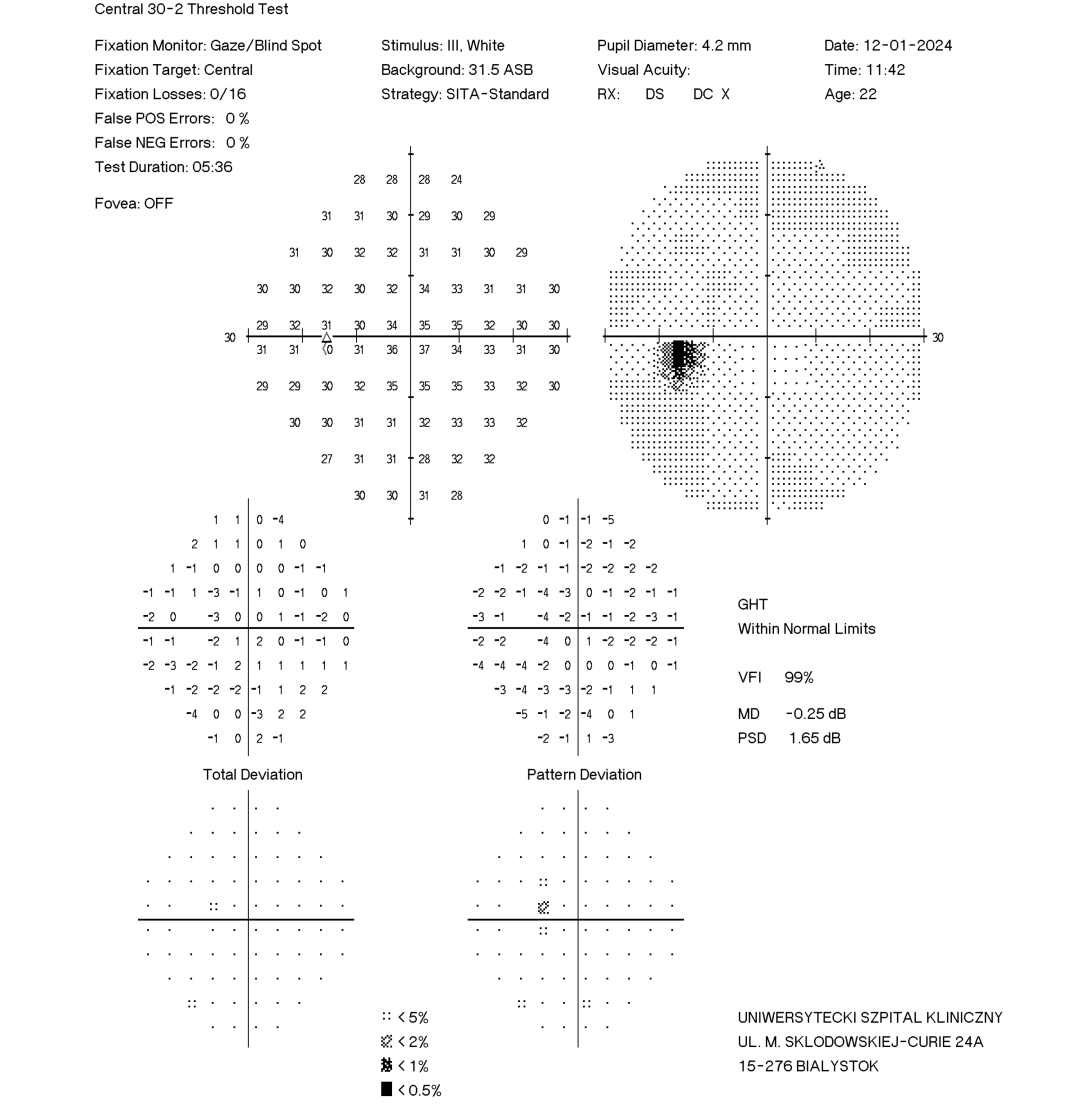
Post-treatment perimetry of the left eye of case 2 (after treatment).

After the completion of 10 HBOT sessions, the patient reported complete resolution of symptoms. The local condition of the left eye remained unchanged since the previous visit, but follow-up OCT revealed a significant reduction in the swelling of the inner retinal layers. In addition to the morphological improvement, a functional enhancement was observed on perimetric examination of the left eye, with the complete disappearance of scotomas (MD = −0.25 dB compared to the pre-HBOT MD = −2.68 dB; Figure 9).

**Figure 10:**
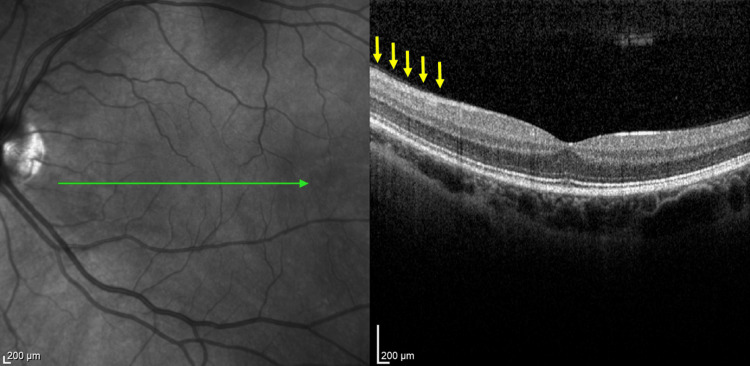
OCT image of patient 2 after 10 HBOT sessions. Yellow arrows indicate the post-treatment area of the previous inner retinal layers swelling corresponding to Figure 7. OCT, optical coherence tomography; HBOT, hyperbaric oxygen therapy.

At the three-month post-HBOT follow-up visit, the patient denied the recurrence of any ocular complaint. Both fundus and OCT examinations showed a reduction of the retinal edema in the left eye (Figure 10). The patient did not report any adverse effects or complications during or after HBOT.

Case 3

**Figure 11:**
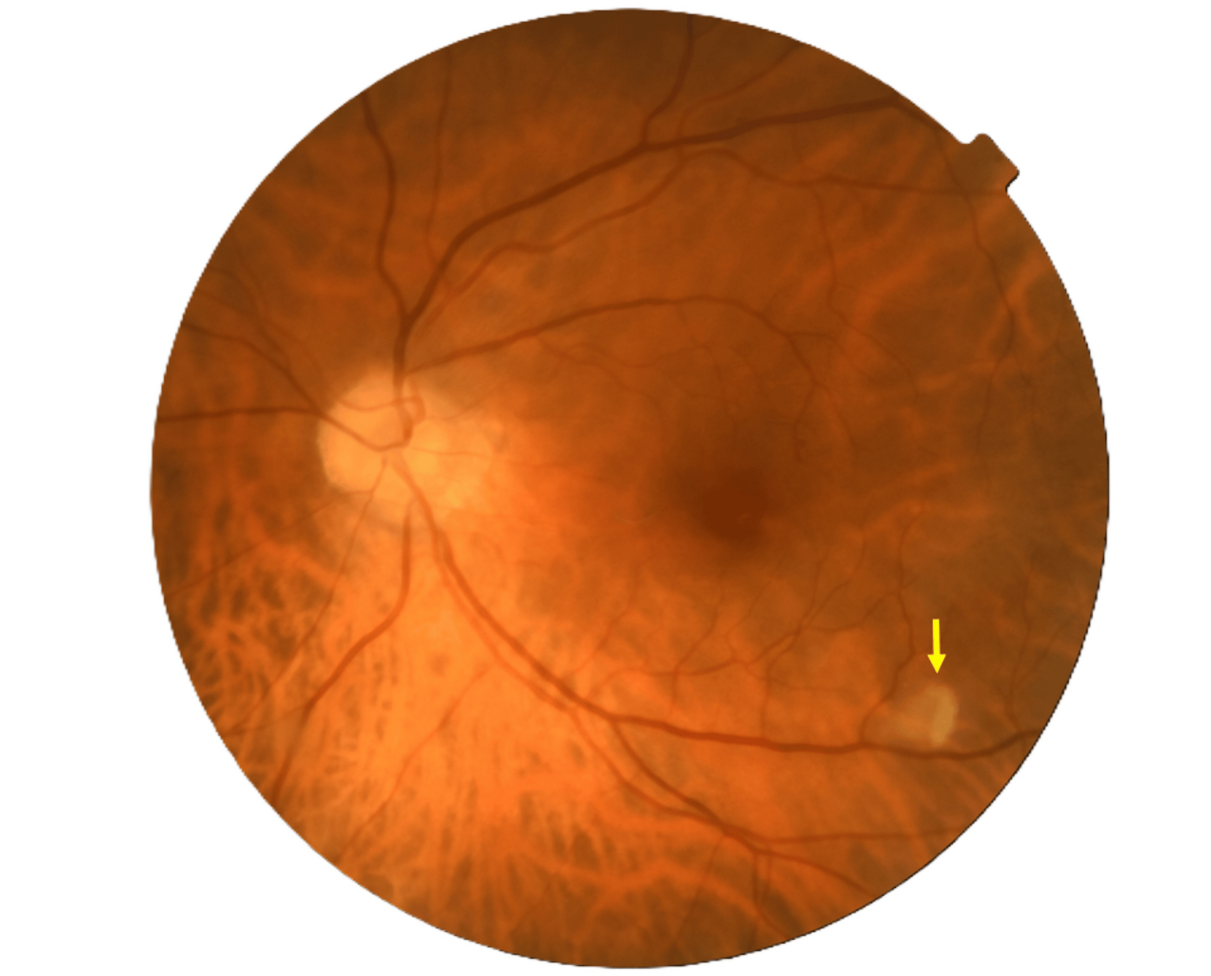
Pretreatment fundus image of case 3 at the initial visit (before treatment). The yellow arrow indicates the “cotton wool spot”.

**Figure 12:**
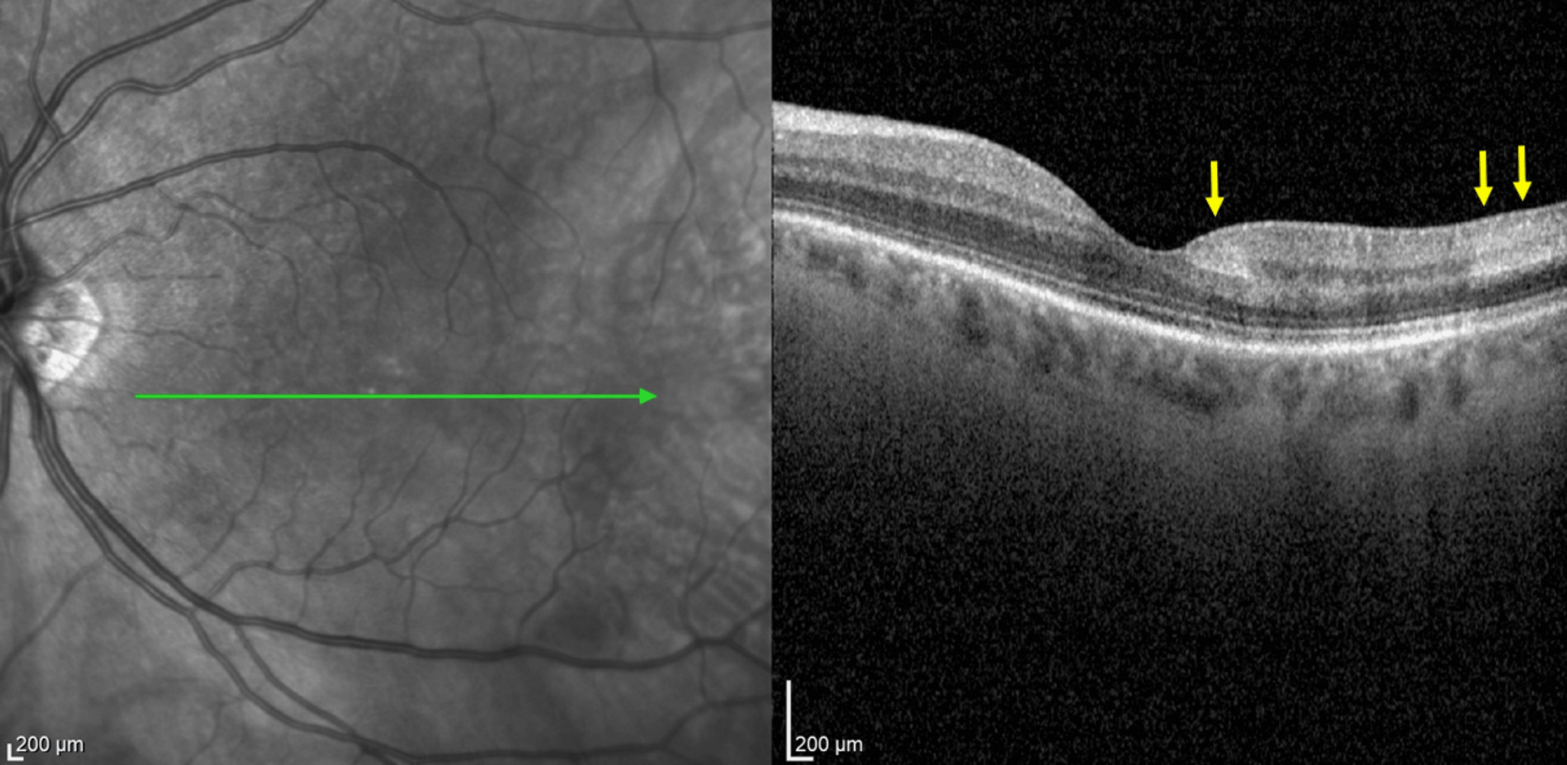
Macula of the left eye with hyperreflectivity of the inner nuclear layer and RNFL. Yellow arrows indicate the area of hyperreflectivity of the inner nuclear layer and RNFL. RNFL, retinal nerve fiber layer.

A 60-year-old woman presented to the hospital ambulatory with a severe headache and a visual field defect in the nasal quadrant of the left eye. The symptoms occurred during the morning hours following the embolization of an internal carotid artery aneurysm in the brain. She had a history of well-controlled hypertension and was on long-term treatment with blood pressure-lowering medication (captopril 12.5 mg once daily). CT of the head revealed no intracranial hemorrhage, and neurological examination revealed no significant neurological impairment. Negative results for CRP and ESR ruled out an inflammatory process (Table 4). The BCVA of the right eye was 1.0, and that of the left eye was 0.7 (Snellen notation), while the IOPs were 14.5 and 16.0 mmHg for the right and left eyes, respectively. No impairment in color discrimination was observed with the Ishihara plates. A bilateral anterior segment examination revealed no pathological changes. Fundus examination of the left eye revealed a flat, pink optic disc with clear borders. In the posterior pole, we observed a 0.5 DD area of subtle retinal whitening with an associated "cotton wool spot" above the inferior arcade temporally from the fovea (Figure 11). OCT (Heidelberg Engineering OCT Spectralis®) of the left macula visualized hyperreflectivity of the inner nuclear layer and RNFL temporally from the fovea and above the inferior arcade (Figure 12). Angio-OCT examination of the left macula revealed decreased vascular density in the retinal intermediate choroid plexus. We diagnosed BRAO in the left eye based on these findings. We urgently referred the patient for HBOT and prescribed pentoxifylline 400 mg orally three times daily. The first HBOT session in a daily regimen (1.5-hour HBOT per day at a compression of 2.5 ATA) commenced approximately five hours after symptom onset.

**Table 4:** Laboratory tests results (case 3). CRP, C-reactive protein; ESR, erythrocyte sedimentation rate.

Laboratory test	Test results	Reference range
CRP	2.2 mg/L	0.0-10.0 mg/L
ESR	20 mm/2 h	<24 mm/2 h

**Figure 13:**
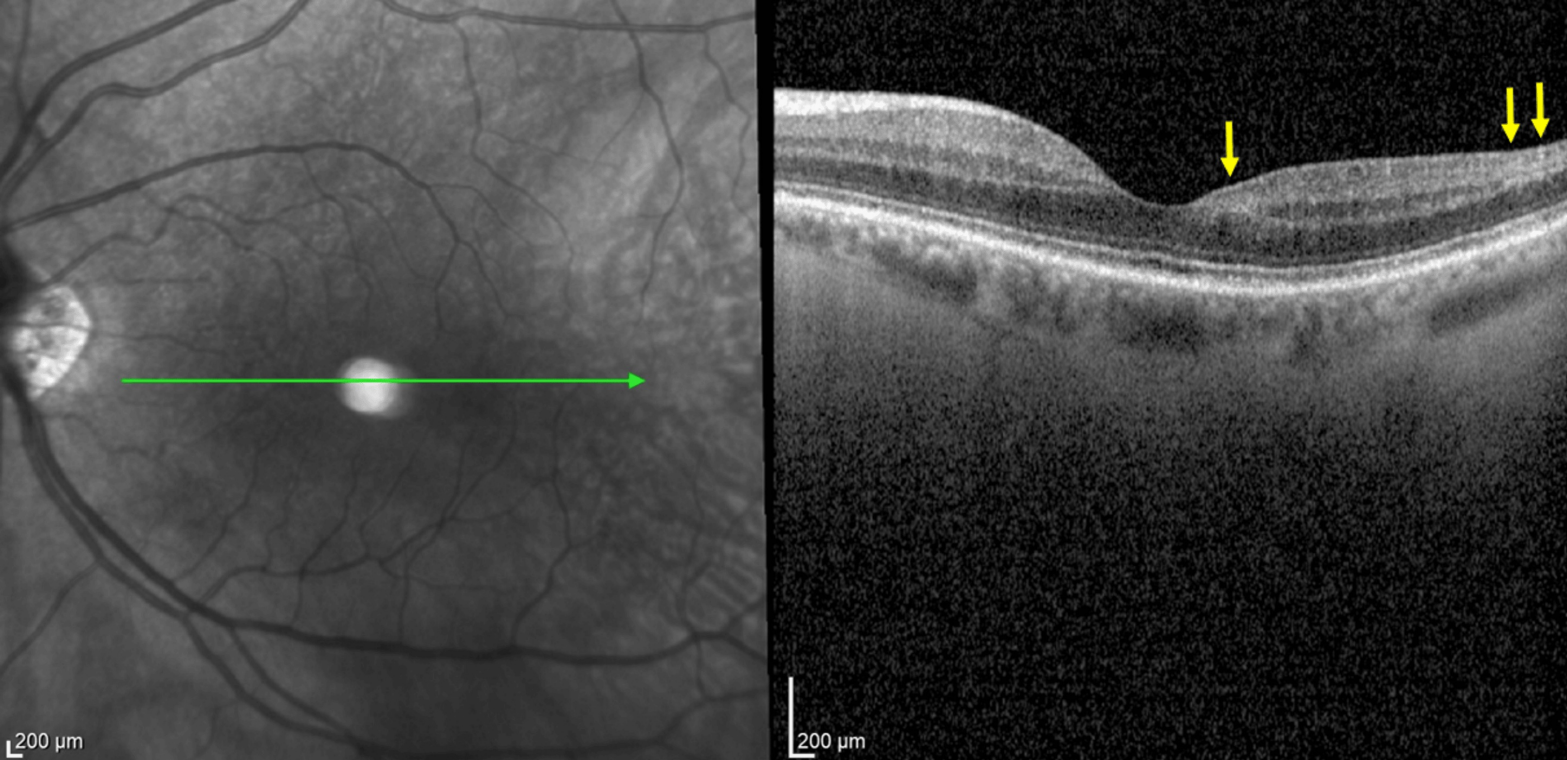
OCT image of case 3 after 10 HBOT sessions. Yellow arrows indicate the post-treatment area of the previous inner nuclear layer and RNFL hyperreflectivity corresponding to Figure 12. HBOT, hyperbaric oxygen therapy; OCT, optical coherence tomography.

The patient reported improved vision in the left eye after the seventh session of HBOT. The BCVA and IOP of the left eye were 1.0 and 15.5 mmHg, respectively. The local conditions of the anterior segment and fundus of the left eye did not differ from those observed during the previous examination. At the next ophthalmological follow-up, one day after completing 15 HBOT sessions, the patient reported complete resolution of symptoms. Fundus imaging of the left eye showed resolution of swelling of the RNFL at the inferior arcade. Follow-up OCT examination of the macula revealed temporal thinning of the inner retinal layers from the fovea (Figure 13). The patient denied any recurrence of ocular complaints at a subsequent follow-up visit approximately two months after hyperbaric therapy. No HBOT-related adverse events were reported.

Written informed consent was obtained from all patients prior to HBOT. The treatment was administered in accordance with the tenets of the Declaration of Helsinki and approved by the Bioethics Committee of the Medical University of Bialystok (APK: APK.002.425.2022). All patients were followed up until June 2025 without long-term complications (Table 5).

**Table 5:** Summary of the HBOT applied to cases 1, 2, and 3. ATA, atmospheric absolute; BCVA, best corrected visual acuity; HBOT, hyperbaric oxygen therapy.

Case	Time of the initial HBOT session after symptom onset	BCVA pre-HBOT	BCVA post-HBOT	Total number of HBOT sessions	Conditions of each HBOT session
Case 1	36 h	1.0	1.0	13	1.5 h in 2.5 ATA
Case 2	48 h	1.0	1.0	10	1.5 h in 2.5 ATA
Case 3	5 h	0.7	1.0	15	1.5 h in 2.5 ATA

## Discussion

The cases described above demonstrate the universal benefit of HBOT in preserving retinal viability and, consequently, functional BCVA during BRAO, which has several etiologies. Animal model studies have indicated that retinal ischemia during RAO causes retinal damage as early as 100 minutes and results in irreversible retinal impairment 240 minutes after the onset of the vascular incident [4,20].

The Undersea and Hyperbaric Medical Society has issued a level IIB recommendation in favor of the utilization of HBOT in cases of CRAO [21]. This recommendation was further substantiated by the clearance issued in 2021 by the US Food and Drug Administration for hyperbaric chambers as safe and effective devices for the treatment of RAO [11]. The rationale behind HBOT for RAO is that while the outer retina is nourished by the ciliary arteries via the choriocapillaris (CC), the inner retina receives its vascular supply from the CRA. Abrupt vision loss in patients with CRAO results from cell death in the inner retinal layer. However, the outer layers tend to remain relatively unaffected, allowing oxygen from the CC to permeate into the inner layers of the retina in sufficient amounts to maintain retinal function and recover vision [9]. Retinal vessels are terminal in nature, lacking anastomoses between the retinal and choroidal vessels unless a cilioretinal artery is present. In the event of RAO, retinal cells can remain viable for several hours because of the oxygen content within the vitreous chamber and heightened anaerobic glycolysis in the visual cells [9]. In certain instances, normobaric hyperoxia may be sufficient, but HBOT may be necessary in others. Despite normoxic conditions, the CC remains responsible for the majority (60%) of retinal oxygen supply, irrespective of whether hyperoxic conditions can adequately fulfill retinal oxygen demand through the CC.

In the present case series, patients underwent HBOT at 5, 36, and 48 hours after the onset of ocular symptoms. Despite the relatively prolonged period of HBOT implementation following the onset of symptoms in our patients, the ischemic effect of BRAO/HRAO, which is restricted to a specific area of the retina, compared with CRAO, may have contributed to the recovery of full BCVA and the complete reversal of the reported visual field defects following treatment. This finding highlights the potential to extend the therapeutic window for these types of vascular incidents. However, given the limited number of publications on this topic and the lack of prospective population-based studies, a specific time frame cannot be defined at present. Similar findings were reported in the study by Schmidt et al. [17], where a statistically better improvement in BCVA was observed for 14 patients with BRAO after seven sessions of HBOT compared with the control group (which received only conservative treatment). The BCVA in the HBOT group improved from 0.18 ± 0.19 to 0.69 ± 0.29, while that in the control group improved from 0.23 ± 0.19 to 0.32 ± 0.23. Moreover, the mean duration from the onset of ocular symptoms to the first session of HBOT was 19.39 ± 27.49 hours. Furthermore, individual case reports presented beneficial effects of HBOT alone and in combination with transluminal Nd:YAG laser embolysis on visual acuity [17,18,22]. Additionally, Hsieh and Lee [19] described the case of a patient affected by simultaneous BRAO and BRVO who experienced a return of BCVA to 1.0 in the affected eye five months after the termination of hyperbaric therapy. This opens up a discussion on the wider use of HBOT for other types of vascular ophthalmic conditions.

Murphy-Lavoie et al. [23] proposed an algorithm designed to address the treatment of CRAO with HBOT. The algorithm primarily targets patients who have ophthalmic symptoms within a maximum of 24 hours before presenting to a reference center. The proposed approach involves initiating HBOT immediately at the highest possible fraction of inspired oxygen once daily and meticulously monitoring the recanalization process of the visualized vessel, with the possibility of modifying the treatment strategy based on clinicomorphological features [23].

A more intensive HBOT regimen was proposed by Chiabo et al. in a study involving 31 patients with CRAO or BRAO [24]. Statistics from their study indicated slightly better efficacy of therapy [24]. The authors observed an improvement in BCVA of at least −0.3 logMAR after one month in 15 of 31 patients enrolled in HBOT due to an episode of RAO that began no more than seven days before ophthalmologic examination. Moreover, the researchers observed better BCVA improvement in the subgroup of patients with CRAO (52.6%) than in those with BRAO (41.6%), as well as among patients with lower baseline BCVA and those receiving antiplatelet therapy. However, the researchers employed a more aggressive treatment regimen, administering two 90-minute sessions per day for 15 days. This approach, along with the potential duration of the treatment window, highlighted the significance of therapy intensity.

Similar results were reported by Lopes et al. [16], in which 75% and 55.5% of four patients with BRAO and nine patients with CRAO exhibited BCVA improvement of at least −0.3 logMAR from baseline. Of these, 77% received HBOT within 12 hours of symptom onset, with a median of seven sessions [16]. Moreover, the authors highlighted the safety of this therapy. Of the 31 patients who underwent HBOT for RAO, nine showed long-term BCVA improvement (median number of sessions, one to seven) [16]. The patients who were observed to have positive effects of the therapy on BCVA underwent their first session in the hyperbaric chamber less than 10 hours from symptom onset, indicating the need to implement HBOT as early as possible.

The results of the retrospective study by Williamson et al. slightly differed from those of the cited publications [25]. The researchers observed a statistically significant improvement in BCVA (−0.2 logMAR) after HBOT in a group of 17 patients with CRAO, with no corresponding trend in a group of five patients affected by BRAO. Meanwhile, the patients had no irreversible complications related to the therapy. This emphasizes the increasing awareness and availability of hyperbaric therapy, which may result in significantly better treatment outcomes.

Consensus on the timing of HBOT and the number of sessions is lacking. In our study, the initial sessions were beneficial and significantly improved BCVA; however, some studies have reported that patients showed improvement after the seventh session [17]. Due to the small number of patients in our case series, we did not make any assumptions regarding the optimal number of HBOT sessions.

Our research has a few limitations. The patients were observed over a period of two years, during which no permanent side effects of HBOT were detected. However, our observation was primarily based on ophthalmic examination and qualitative information, including medical history analysis and symptoms reported by patients during follow-up visits, with a paucity of quantitative data on potential HBOT toxicity. Breathing oxygen at increased pressure induces alterations in hemoglobin saturation levels, which facilitate the transfer of surplus oxygen to tissues. This mechanism can potentially surpass tissue oxygen requirements, disrupting their capacity to effectively utilize available oxygen. Exposure to elevated oxygen pressure can enhance the delivery of oxygen to body tissues by up to 20 times. Consequently, HBOT increases concentrations of reactive oxygen and nitrogen species in both tissues and blood, which may induce oxidative DNA damage. The long-term consequences of such damage are difficult to predict. To definitively define the role of HBOT in the treatment of RAO, further large, well-defined cohort studies using standardized methodology and carefully considering the unique characteristics of individual patients are needed.

Another issue is that the natural progression of eyes with BRAO is often favorable, as observed by Hayreh et al. [7], with almost all eyes showing improvement over time. Therefore, it is not easy to draw exact conclusions on whether improvement occurred due to HBOT or natural progression. Another challenge is the availability of HBOT. In several countries, the cost of such treatment may not be covered by medical insurance or government funding. Furthermore, logistical challenges must be addressed, as such treatment is not readily available and is expensive. The only way to determine the efficacy of such treatment would be a randomized prospective trial or a case-control study, with the latter being easier and more practical due to the infrequency of BRAO.

## Conclusions

HBOT has beneficial effects on the final visual acuity of patients following an episode of BRAO. It may serve as a rescue treatment for early-stage BRAO, particularly during the period when reperfusion is feasible. Further research involving more diverse groups of patients and randomized controlled trials is necessary to confirm this hypothesis.
